# Drug formulation studies on regulation of BCL-2 family for treatment of autism

**DOI:** 10.1186/1471-2164-15-S2-P38

**Published:** 2014-04-02

**Authors:** Iftikhar A Tayubi, Sayane Shome, Sarosh Hashmi

**Affiliations:** 1 Faculty of Computing and Information Technology, King Abdulaziz University, Rabigh-21911, Saudi Arabia; 2 School of Biosciences and Technology, Vellore Institute of Technology, Vellore-632014, India; 3 Faculty of Computing and Information Technology, King Abdulaziz University, Rabigh-21911, Saudi Arabia

## Background

**Figure 1:**
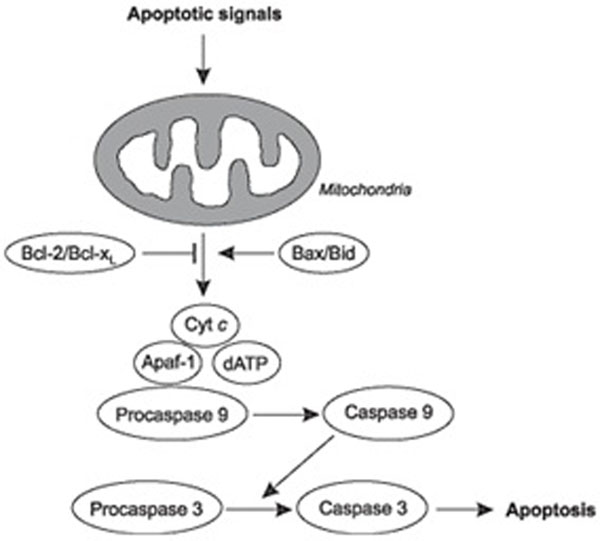
(Left): Regulation of apoptosis pathway by family of BCL-2 proteins

BCL2 and Bax are two members of BCL2 family which is responsible for the regulation of apotosis [1,2]. BCL2 protein duels with its counteracting twin, a partner known as Bax (Figure 1). When Bax is in excess, cells execute a death command. When BCL2 dominates, the program is inhibited and cells survive. The intent is to propose a drug complex which aids in regulation of the BCL2 and Bax levels, aiding in treatment of autism. Navitoclax is known drug responsible for inhibiting BCL2 production for the treatment of cancer. We suggest a drug formulation of navitoclax with a Bax channel locker.

## Materials and methods

**Figure 2:**
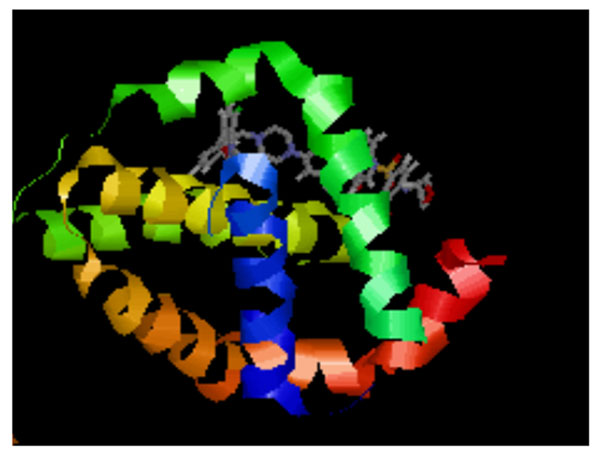
(Top right): BCL-2 protein docked with Navitoclax [PDB Id: 4LXE]

The structures of Navitoclax (Pubchem ID : 923564-51-6) and Bax channel locker (Pubchem ID :335165-68-9) were taken from Pubchem database. Their binding affinity and energies with target components were analysed using Autodock 4.0.The active site residues in the target proteins BCL2 (Figure 2) and BAX were determined by literature studies and using CastP and Sp align software. The admet properties and pharmacological properties of the drug were calculated using Discovery studio software.

## Results

**Figure 3:**
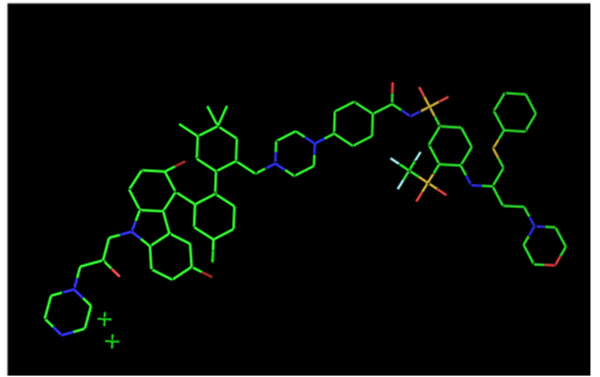
(Bottom right): Chemical structure of the novel designed drug complex

Based on the binding affinities of Navitoclax and Bax Channel Locker with its respective target proteins BCL2 and Bax respectively, the target functional groups to connect the two ligands were shortlisted. It was taken care the responsible target binding sites aren’t affected with the linkage. Four combinations (Figure 3) were created. Their pharmacological properties were predicted insilico to shortlist the best combination. The predicted properties inferred the drug complex to be non-carcinogenic, and non-mutagen and may display moderate irritancy. The novel drug complex can be further modified and analyzed to get drug with lower molecular weight and displaying low to no irritancy.

## Conclusions

The admet and chemical properties suggest there are possibilities to consider the new drug complex designed for further analysis.
